# Development of an evidence-based strategy to assess and manage substance use in oncology patients

**DOI:** 10.1186/1940-0640-10-S2-O12

**Published:** 2015-09-24

**Authors:** Amanda Choflet, Laura Hoofring, Sarah Bonerigo, Lisa Katulis, Omar Mian, Amol Narang, Marian Richardson, Sue Appling

**Affiliations:** 1 Radiation Oncology, Johns Hopkins Hospital, Baltimore, USA; 2 Department of Oncology, Johns Hopkins Hospital, Baltimore, USA; 3 Johns Hopkins University School of Nursing, Baltimore, USA

## Background

This project will empower cancer patients to address their substance use by establishing an evidence-based strategy for identifying and managing substance use during cancer treatment.

The question is: Does the proactive identification and management of substance use in oncology patients improve specific patient outcomes such as quality of life, treatment adherence, and reduction in treatment complications?

A multidisciplinary team identified substance use as a contributing factor in several adverse patient outcomes. Further research revealed that there are currently no evidence-based standards to guide the care of patients with alcohol and illicit substance use (AISU) who are diagnosed with cancer. AISU during cancer treatment significantly worsens quality of life outcomes, including problems with pain, sleep, dyspnea, total distress, anxiety, coping, shortness of breath, diarrhea, poor emotional functioning, fatigue and poor appetite[1-4]. AISU in cancer patients may also cause significant safety risks in the short and long term[5-9] and increased mortality[10]. After completing a literature review and an exhaustive needs assessment, the team developed a program to implement Screening, Brief Intervention, and Referral to Therapy (SBIRT) in the outpatient radiation oncology department. The implementation plan includes motivational interviewing training, clinical pathways, and risk-based intervention toolkits that will incorporate oncology-specific resources.

## Conclusions

AISU in cancer patients is a threat to safety and quality of life, and patients who use substances during cancer treatment represent an opportunity to improve wellness in a vulnerable population. A program that incorporates the use of SBIRT as part of routine patient care should improve both cancer-specific and all-cause patient outcomes and is critical to patient safety.

## Material and methods

Three evidence-based methods will be implemented to address substance use in cancer patients. Inter-professional instructional design will be provided to nurses and resident physicians to prepare front-line staff to implement a screening, brief intervention, and referral to therapy (SBIRT) program. This instruction will consist of SBIRT-focused motivational interviewing training utilizing standardized patients and coaching support. Following this training, staff will implement risk-based clinical pathways based on validated assessment instruments. Finally, oncology-specific toolkits will be implemented, which will include referral instructions and resources, oncology-specific risk sheets, and validated SBIRT educational items.

## Results

Nurses and residents will complete surveys regarding their knowledge, skills, and attitudes. Screening, brief intervention, and referral rates will also be gathered retrospectively.
